# Effectiveness of Repair of Aortic Coarctation in Neonates: A Long-Term experience

**DOI:** 10.1007/s00246-021-02685-z

**Published:** 2021-08-02

**Authors:** Chiara Minotti, Manuela Scioni, Biagio Castaldi, Alvise Guariento, Roberta Biffanti, Giovanni Di Salvo, Vladimiro Vida, Massimo A. Padalino

**Affiliations:** 1 Pediatric Cardiology, Department of Women’s and Children’s Health, University of Padova, Via Giustiniani 2, 35128 Padova, Italy; 2 Department of Statistical Sciences, University of Padova, Padova, Italy; 3 Pediatric and Congenital Cardiac Surgery, Department of Cardiothoracic and Vascular Sciences and Public Health, University of Padova, Padova, Italy; 4 Division of Cardiovascular Surgery, Department of Surgery, Hospital for Sick Children, Toronto, Canada

**Keywords:** Aortic coarctation, Neonates, Surgical repair, Outcome

## Abstract

To evaluate early and long-term results of surgical treatment of aortic coarctation (CoAo) in neonates. This is a retrospective clinical review of neonates with CoAo, who underwent surgery between 1995 and 2019. Data were retrieved from our institutional database, to identify preoperative and postoperative characteristics. Statistical analysis was performed by means of relative risk ratio and Cox and logistic multivariate analysis. 218 consecutive neonates (M/F: 129/89, median age 11 days, IQR 7–17 days) were included; 202 (92.7%) had a left thoracotomy; 178 underwent extended end-to-end anastomosis (EEEA, 81.6%). Hypoplastic aortic arch (HAA) was present in 102 patients (46.8%); complex cardiac anomalies in 85 (39%). Significant postoperative complications occurred in 20 (9.2%). Thirty-day mortality was 2.3% (most in complex types). At a median follow-up of 10.4 years (IQR 5.6–15.0 years; FU completeness 95.9%), there were 8 late deaths (3.7%), all associated to complex CoAo. Among 196 survivors, 177 (93.2%) were in NYHA class I; re-interventions on aortic arch occurred in 9.2% (2.0% were surgical). Freedom from mortality and re-intervention on aorta at 10 years were 94.3% and 96.7%, respectively. Surgical repair of CoAo in newborns without CPB in our series was safe and low-risk, with excellent early and late outcomes.

## Introduction

Aortic coarctation (CoAo) is a common congenital heart disease (CHD), occurring in approximately 4 out of 10,000 live births [1, 2], which may be isolated or complex, when associated with other CHDs, such as a bicuspid aortic valve (BAV), ventricular septal defect (VSD), and hypoplastic aortic arch (HAA) [3]. In particular, HAA repair in either isolated or complex aortic coarctation may be associated with significant morbidity [4, 5].

Surgical planning is influenced by the need for concomitant repair of associated CHD [6]. In particular, the presence of HAA can be determinant for choosing the surgical approach (i.e., sternotomy vs. thoracotomy) or use of cardio-pulmonary bypass (CPB), deep hypothermic circulatory arrest (DHCA) [7], or selective antegrade cerebral perfusion (ACP) [5, 8].

Since the first report of the end-to-end anastomosis (EEA) by Crafoord in 1945 [9], several techniques have been proposed, such as aortic isthmoplasty [10], subclavian flap aortoplasty [11], extended end-to-end anastomosis (EEEA) [12], end-to-side anastomosis (ESA), and patch/conduit repair. Currently, the optimal surgical approach for CoAo repair in neonates, especially when associated with HAA, is controversial. Undoubtedly, an EEEA through a thoracotomy avoids CPB and the risk of adverse events (AE) related to DHCA or selective ACP [6, 13, 14]. However, a possible disadvantage is leaving the proximal HAA untreated, with a potential need of a future re-intervention.

Numerous studies have explored postoperative outcomes [8, 14–16]. Nevertheless, they had a relatively short follow-up, were limited to one surgical approach, or included patients undergoing surgical repair at different ages.

In this study, we reviewed our experience with neonatal repair of CoAo, to assess early and late morbidity, mortality, and re-intervention rate, with particular attention to late clinical outcomes.

## Methods

This is a single-center, retrospective clinical study including all consecutive neonates (< 30 days of age) undergoing surgical repair from January 1995 to December 2019. A review of medical records was approved by our Hospital Committee on clinical investigation (4451/AO/18). Individual patients were not identified, and the need for patient consent was waived. Demographic, operative, and short-term outcomes included preoperative characteristics, any intervention (surgery or balloon dilation) before CoAo repair (also defined pre-CoAo procedures), associated anomalies, type of repair and surgical approach (thoracotomy vs sternotomy), associated surgical procedures, use of CPB, ACP, or DHCA, onset of major postoperative complications, and early (< 30 days) death. CoAo repair was defined “Complex” when associated with major CHD.

Preoperative echocardiographic images were evaluated by 2 different cardiologists, who were unaware of the original echocardiographic report and clinical outcomes. The dimensions of proximal (PAA between innominate and left carotid-LCA-arteries) and distal aortic arch (DAA, between LCA and left subclavian artery) were measured and indexed by body surface area; we defined HAA when *Z*-scores of PAA and/or DAA were < than − 3, as elsewhere stated [15].

Follow-up (FU) data included clinical status (NYHA class); late (> 30 days) death, re-interventions, surgical and/or catheterization procedures, either cardiac or specifically on the previously repaired aortic site; arterial hypertension requiring medical treatment; aortic aneurysm; aortic valve dysfunction. All these events might have occurred in the same patient. In particular, clinical FU evaluation included a complete 2D Echocardiography to evaluate the peak and medium pressure gradient and the presence of diastolic run-off at the isthmus level with continuous Doppler and abdominal aorta pulsed wave Doppler to study the wave shape and the presence of diastolic tail. A recoarctation was defined as the presence of a superior-inferior limb arterial pressure gradient > 20 mmHg at rest, with or without a mean isthmic pressure gradient > 20 mmHg, and a diastolic tail in pulsed wave Doppler in the abdominal aorta.

### Surgical Technique

Most commonly, on right lateral decubitus, a left mini-thoracotomy was made in the 4th intercostal space. After lung retraction, the stenotic aortic isthmus was exposed. The ductus was suture-ligated and resected, with extensive mobilization of the distal aorta (at least 10 mm below the isthmus). The PAA was extensively mobilized until the LCA or to the innominate artery, to enhance adequate exposure of the entire PAA to the ascending aorta. The aortic isthmus was resected, removing all residual ductal tissue, to avoid late scar tissue retraction. The proximal incision was extended in the concavity of the aortic arch to the origin of the LCA or the innominate artery. Subsequently, both segments were re-approximated in a beveled fashion, and the anastomosis was performed with a continuous 7.0 prolene suture. Repair was considered optimal if the residual upper-lower limbs pressure gradient is ≤ 10 mm Hg.

### Statistical Analysis

Quantitative variables were presented as median ± interquartile range (IQR), while categorical variables were reported as frequency and percentage. A bivariate analysis was performed to assess the effect of a single predictive variable on outcomes; the association with outcomes as early and late death, any re-intervention on aorta, and hypertension, and each predictive variable was measured using the relative risk (RR) and related 95% confidence interval (CI). Risk factors were gender, age class (< 15 days, ≥ 15 days) at surgery, urgent surgery, BAV, hypoplastic LV/single ventricle, pre-CoAo procedures, HAA, complex CoAo, EEEA vs other procedures, CPB, thoracotomy, and associated surgical procedures.

Survival analysis was performed by Kaplan–Meier curves and log-rank test. Multivariate models were applied using Cox proportional hazards for outcomes (death, re-intervention on aorta). Significant risk factors in the bivariate analysis were included in the multivariate models. Multivariate logistic regression was performed to analyze systemic hypertension. The significance level was set at 0.05, and all tests were two-tailed. The inverse probability of treatment weighting (IPTW) was applied to minimize the fact that the surgical procedure was not assigned randomly to the patients, and it was based on the covariates mentioned above. Data were analyzed using R version 2.6.2.

## Results

We included 218 consecutive neonates (M/F: 129/89), with a median age at surgery of 11 days (IQR 7–17 days). CoAo was isolated in 133 (61.0%); 102 (46.8%) had HAA. All preoperative data are listed in Table 1. Most patients underwent EEEA (178, 81.6%). Complete preoperative echocardiographic data were available in 40 patients. Median PAA Z-score was −4.1 (IQR − 6.1 to − 2.7), while median DAA Z-score was − 3.2 (IQR − 4.1 to − 2.2). A left lateral thoracotomy was used in 202 patients (92.7%). Associated procedures were performed in 61 patients (27.9%). Prior to CoAo repair, 8 patients required intervention (balloon dilation in 6, pulmonary artery banding and atrioseptostomy in 2, respectively). Significant postoperative complications occurred in 20 patients (9.2%, Table 2). There were no early reoperations. Thirty-day mortality was 2.3% (5 patients, 3 of whom with complex CoAo, after repair on CPB). All intraoperative and postoperative data are summarized in Table 2.

**Table 1:** Preoperative data

	Total: 218	Isolated CoAo type: 133 (61.0)	Complex CoAo type: 85 (39.0)	*p* value
Male (*n*,%)	129/218 (59.2)	81/129 (62.8)	48/129 (37.2)	*p* = 0.516149
Median age at surgery	11 days (IQR 7–17)	11 days (IQR 8–16)	12 days (IQR 7–18)	NA
Age category (*n*,%)				
< 15 days	142 (65.1)	93/142 (65.5)	49/142 (34.5)	*p* = 0.195417
≥ 15 days	76 (34.9)	43/76 (56.5)	33/76 (43.5)	
HAA (*n*,%)	102 (46.8)	41/102 (40.0)	61/102 (60.0)	***p* = 0.00001**
BAV (*n*,%)	65 (29.8)	37	28	*p* = 0.420099
Hypoplastic LV (*n*,%)	11 (5.1)	0/11 (0)	11/11(100)	NA
Single ventricle (*n*,%)	1 (0.5)	0/1 (0)	1/1 (100)	NA
Pre-CoAo surgical intervention (*n*,%)	2 (0.9)	1/2 (50)	1/2 (50)	*p* = 1
Pre-CoAo balloon dilation (*n*,%)	6 (2.8%)	6/6 (100)	0/6 (0)	NA

Significant *p* value is given in bold

*BAV* bicuspid aortic valve, *CoAo* aortic coarctation, *HAA* hypoplastic aortic arch, *LV* left ventricle, *NA* not applicable

**Table 2:** Early and late outcomes

	Total 218	Isolated CoAo type:133 (61.0)	Complex CoAo type: 85 (39.0)	*p* value
Surgical intervention				
EEEA (*n*,%)	178 (81.6)	104/178 (58.4)	74/178 (41.6)	*p* = 0 .099148
EEA (*n*,%)	35 (16.1)	18/35 (51.4)	17/35 (48.6)	*p* = 0.204661
Other (*n*,%)^a^	5 (2.3)	4/5 (80)	1/5 (20)	*p* = 0.378414
Operative approach				***p* = 0 .000036**
Lateral thoracotomy (*n*,%)	202 (92.7)	131/202 (64.8)	71/202 (35.2)	
Median sternotomy and CPB (*n*,%)	16 (7.3)	2/16 (12.5)	14/16 (87.5)	
Associated surgical procedures (*n*,%)	61 (27.9)	16/61 (26.2)	45/61 (73.8)	***p* < 0.00001**
Urgent procedure (*n*,%)	23 (10.5)	11/23 (47.8)	12/23 (52.2)	*p* = 0.170497
Early outcomes				
Postoperative complications (*n*,%)	20 (9.2)	9/20 (45)	11/20 (55)	*p* = 0.123491
Heart failure (*n*)	5	3/5 (60)	2/5 (40)	
Respiratory failure (*n*)	3	1/3 (33.3)	2/3 (66.7)	
Infections/sepsis (*n*)	3	0/3 (0)	3/3 (110)	
Arrhythmias (*n*)	2	2/2 (100)	0/2 (0)	
Renal failure (*n*)	2	1/2 (50)	1/2 (50)	
Pulmonary hypertension (*n*)	1	0/1 (0)	1/1 (100)	
Other (*n*)	4	2/4 (50)	2/4 (50)	
Need for re-intervention (*n*,%)	0 (0)			NA
Thirty-day mortality (*n*,%)	5 (2.3)	2/133 (1.5)	3/85 (3.5)	*p* = 0.32984
Late outcomes				
Alive with follow-up (*n*,%)*:*	196 (89.9)	125/196 (64.7)	71/196 (35.3)	***p* = 0.012433**
Lost to follow-up (*n*,%)*:*	9 (4.1)	6/9 (66.6)	3/9 (33.4)	*p* = 0.722287
Median follow-up time (years, IQR)	10.4 (5.6–15.0)	10.6 (6.7–16.5)	10.2 (1.3–14.9)	*p* = 0.08726
Late mortality (*n*,%)*:*	8 (3.7)	0	8	***p* = 000,757**
Overall mortality (*n*,%)*:*	13 (6.0)	2	11	***p* = 0.000505**
Re-intervention on aorta (*n*,%)	18 (9.2)	8/18	10/18	*p* = 0.132486
Balloon dilation (*n*,%)	14 (7.4%)	7	7	*p* = 1
Surgical re-intervention (*n*,%)	4 (1.8%)	1	3	*p* = 0.136135
NYHA class				
I (*n*,%)	183 (93.4)	120/183 (65.6)	63/183 (34.4)	***p* = 0.001579**
II (*n*,%)	12 (6.1)	5/12 (41.6)	7/12 (58.4)	*p* = 0.166061
III (*n*,%)	0 (0)	0 (0)	0 (0)	NA
IV (*n*,%)	1 (0.5)	0/1 (0)	1/1 (100)	NA
Anti-hypertensive treatment (*n*,%)	18 (9.2)	2/18 (11.1)	16/18 (88.8)	***p* < 0.00001**

Significant *p* values are given in bold

*CPB* cardio-pulmonary bypass, *CoAo* aortic coarctation, *EEEA* extended end-to-end anastomosis, *EEA* end-to-end anastomosis, *NA* not applicable, *NYHA* New York Heart Association

^a^Other: aortic arch reconstruction with patch plasty and termino-lateral anastomosis

### Follow-Up

Late clinical outcomes are summarized in Table 2. At a median FU of 10.4 years (IQR 5.6–15.0 years; completeness 95.9%), there were 8 (3.7%) late deaths in the complex group. Among 196 survivors (89.9%), re-interventions on aortic arch occurred in 18 (9.2%), but only 4 patients (1.8%) needed a surgical reoperation (3 in complex CoAo), while the remaining were effectively treated with balloon angioplasty. Among survivors, 183 (93.4%) were in NYHA class I and 18 (9.2%) were on anti-hypertensive medical treatment. However, all patients were presented at FU with a normal abdominal aorta pulsed wave Doppler ultrasound and a superior-inferior limbs arterial pressure < 20 mmHg at rest.

Bivariate analysis (Table 3a) showed that the presence of hypoplastic LV, HAA, or complex CoAo significantly affected survival. Conversely, patients undergoing EEEA had a significantly lower risk of re-intervention, showing better outcomes (RR 0.385, *p* = 0.048) (Table 3b)**.** Pre-CoAo correction procedures, any surgical technique other than EEA and EEEA, use of CPB, complex CoAo, associated surgical procedures, and urgent operation were significant risk factors for late hypertension (Table 3c).

**Table 3:** Bivariate analysis

	RR	Lower	Upper	*p*-value	*n*
a. RR for early or late death					
Sex (female)	2.319	0.784	6.858	0.148	218
Age ≤ 15gg	0.856	0.290	2.527	0.771	218
Bicuspid aortic valve	0.428	0.098	1.877	0.353	218
Hypoplastic LV	16.13	6.52	39.92	< 0.001	218
HAA	13.65	1.81	103.14	< 0.001	218
Isolated vs complex	8.606	1.955	37.875	< 0.001	218
Surgical procedure					
EEEA	0.590	0.168	2.070	0.421	218
Other than EEA/EEEA	2.333	0.297	18.309	0.427	
Sternotomy and cardio-pulmonary bypass	3.788	1.157	12,394	0.059	218
Thoracotomy	0.264	0.081	0.864	0.059	218
Associated surgery	1.609	0.548	4.725	0.360	218
Urgent procedure	0.782	0.107	5.718	1	218
b. RR for re-intervention on aorta					
Sex (female)	1.263	0.522	3.058	0.619	196
Age class at surgery (≤ 15 gg)	1.350	0.503	3.628	0.613	196
Bicuspid aortic valve	2.015	0.840	4.834	0.122	196
Hypoplastic LV	–	–	–	1	196
Pre-CoAo procedure	2.506	0.98’	6.406	0.079	196
HAA	1.879	0.804	4.393	0.159	196
Isolated vs complex	2.106	0.871	5.095	0.124	196
Surgical procedure					
EEEA	0.385	0.156	0.949	0.048	196
Other than EEA/EEEA	0	0	–	0.553	
Sternotomy and cardio-pulmonary bypass	1.917	0.497	7.386	0.303	176
Thoracotomy	0.522	0.135	2.011	0.303	176
Associated surgical procedures	1.591	0.650	3.896	0.411	176
Urgent procedure	0	0	Na	0.225	176
c. RR for late onset of Hypertension					
Sex (female)	0.602	0.224	1.621	0.447	194
Age class at surgery (≤ 15 gg)	0.523	0.218	1.256	0.192	194
Bicuspid aortic valve	1.000	0.393	2.544	1	194
Hypoplastic LV	2.809	0.485	16.277	0.323	194
Pre-CoAo procedure	6.679	2.835	15.731	0.003	194
HAA	1.267	0.526	3.055	0.626	194
Isolated vs complex	8.356	2.504	27.888	< 0.001	194
Single ventricle	0	0	Na	1	194
Type of surgical procedure					
EEEA	1.259	0.299	5.306	1	194
Other	11.625	2.713	49.806	0.006	194
Sternotomy and cardio-pulmonary bypass	7.625	3.471	16.749	< 0.001	194
Thoracotomy	0.131	0.060	0.288	< 0.001	194
Associated surgical procedures	4.964	1.959	12.577	< 0.001	194
Urgent procedure	3.365	1.339	8.460	0.024	194

*CoAo* aortic coarctation, *EEA* end-to-end anastomosis, *EEEA* extended end-to-end anastomosis, *HAA* hypoplastic aortic arch, *LV* left ventricle, *RR* risk ratio

Logistic regression confirmed that pre-CoAo procedures, urgent repair, and sternotomy were associated with late onset of arterial hypertension (Table 4). After IPTW, the logistic regression confirmed that the type of surgical procedure was affecting signfiicantly the risk for late arterial hypertension (Table 5). Also, in the subgroup with available measurements, a *Z*-score < − 3 of PAA, DAA, and isthmus did not result statistically significant for development of late arterial hypertension (*p* = 0.459, *p* = 0.310, *p* = 0.6, respectively).

**Table 4:** Logistic regression for hypertension

	Estimate	Std error	*z*-value	*p*-value	*n*
Pre-CoAo correction Interventions	2.7945	0.9318	2.999	0.00271	194
Associated surgical procedures	0.9487	0.6619	1.433	0.15181	194
Urgent surgery	1.7193	0.7488	2.296	0.02166	194
Complex CoAo	1.1632	0.7613	1.528	0.12654	194
Sternotomy	2.1531	0.8996	2.393	0.01669	194
Type of repair EEEA vs EEA	1.3673	1.0635	1.286	0.19856	194
Type of repair other vs EEA	2.8298	1.5879	1.782	0.07473	194
Intercept	−3.4603	1.4151	−2.445	0.01447	194

*EEA* end-to-end anastomosis, *EEEA* extended end-to-end anastomosis, *Std* standard, *RR* risk ratio

**Table 5:** Logistic regression for hypertension after inverse probability weighting

	Estimate	Std error	*z*-value	*p*-value	*n*
Type of repair EEEA vs other	0.5579	0.6623	0.842	0.4006	194
Intercept	−5.4696	0.8034	−6.808	< 0.001	194

*EEA* end-to-end anastomosis, *EEEA* extended end-to-end anastomosis, *Std* standard, *RR* risk ratio

Multivariate analysis at Cox’s regression model (Table 6) showed that only a hypoplastic LV was a significant risk factor for mortality (HR 18.74, *p* < 0.001), while HAA was not significantly associated with reoperation (*p* = 0.363). As far as risk for late re-intervention on aorta is concerned, we included control variables other than EEEA (HAA, thoracotomy vs sternotomy approach, complex vs simple CoAo) in the multivariate Cox model, and we observed a tendency of EEEA to protect from re-intervention (*p* = 0.09). However, after IPTW, the type of surgical procedure (EEEA vs EEA and other) was not significantly affecting mortality and re-intervention on aorta (Table 7).

**Table 6:** Multivariate analysis: Cox’s regression model

Variables	Outcomes HR (CI 95%) *p* value	
	Death	Re-intervention on aorta
Hypoplastic LV	18.74 (5.43–64.7) *p* < 0.001	–
Thoracotomy	0.39 (0.10–1.6) p = 0.184	0.75 (0.16–3.5) *p* = 0.718
HAA	7.68 (0.94–62.8) *p* = 0.057	1.68 (0.55–5.2) *p* 0.363
Complex CoAo	2.12 (0.41–11.0) *p* = 0.371	1.85 (0.64–5.4) *p* = 0.259
EEEA vs other techniques	–	0.43 (0.6–1.2) *p* = 0.093

*EEEA* extended end-to-end anastomosis, *HR* hazard ratio, *LV* left ventricle, *HAA* hypoplastic aortic arch, *CoAo* aortic coarctation

**Table 7:** Multivariate analysis: Cox’s regression model after inverse probability weighting

Variables	Outcomes HR (CI 95%) *p* value	
	Death	Re-intervention on aorta
EEEA vs other techniques	0.810 (0.2, 2.9) *p* = 0.745	0.47 (0.16–1.4) *p* = 0.163

*EEEA* extended end-to-end anastomosis, *HR* hazard ratio, *LV* left ventricle, *HAA* hypoplastic aortic arch, *CoAo* aortic coarctation

**Fig. 1:**
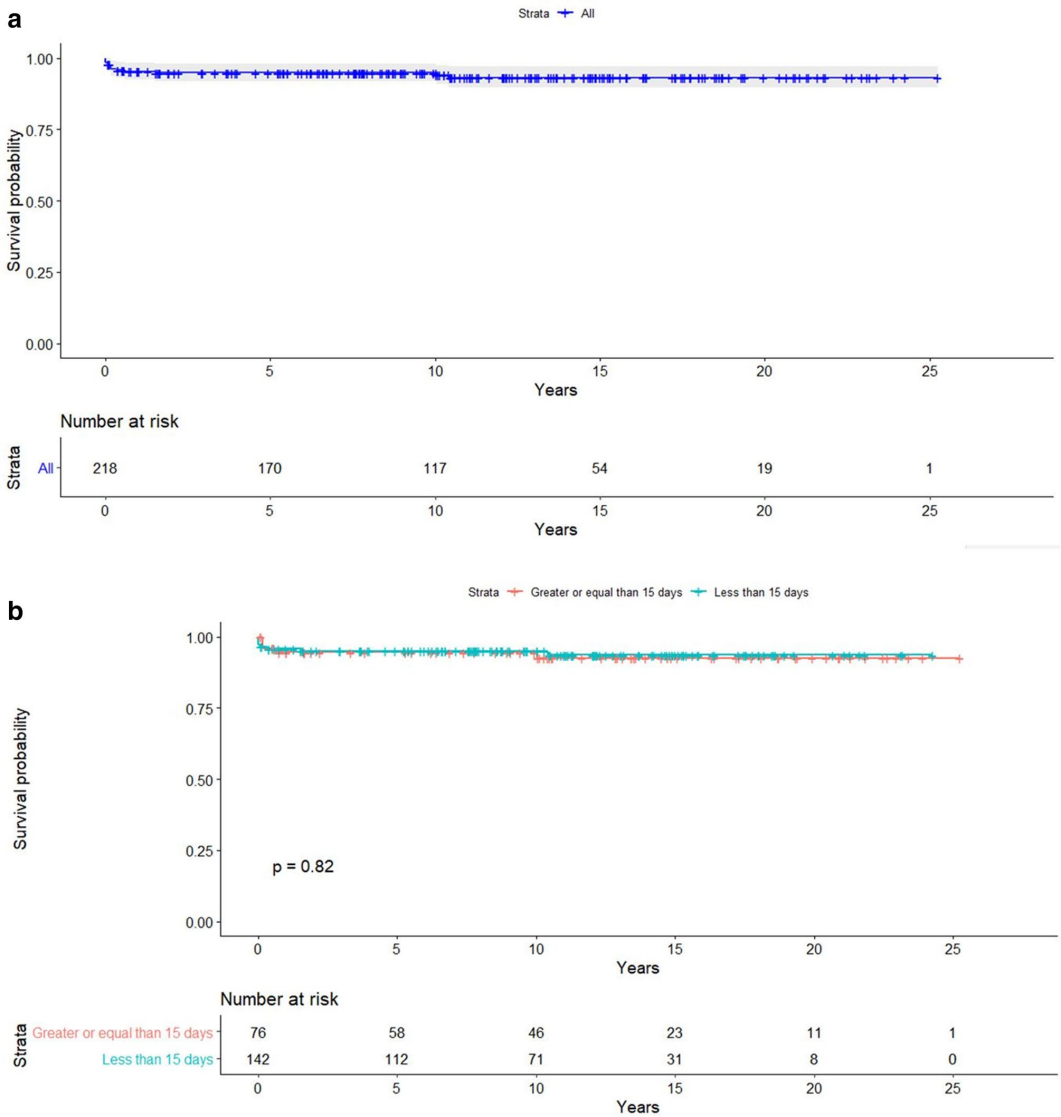
Kaplan–Meier survival plot, showing freedom from mortality, overall (**a**) and according to age subgroup (**b**)

**Fig. 2:**
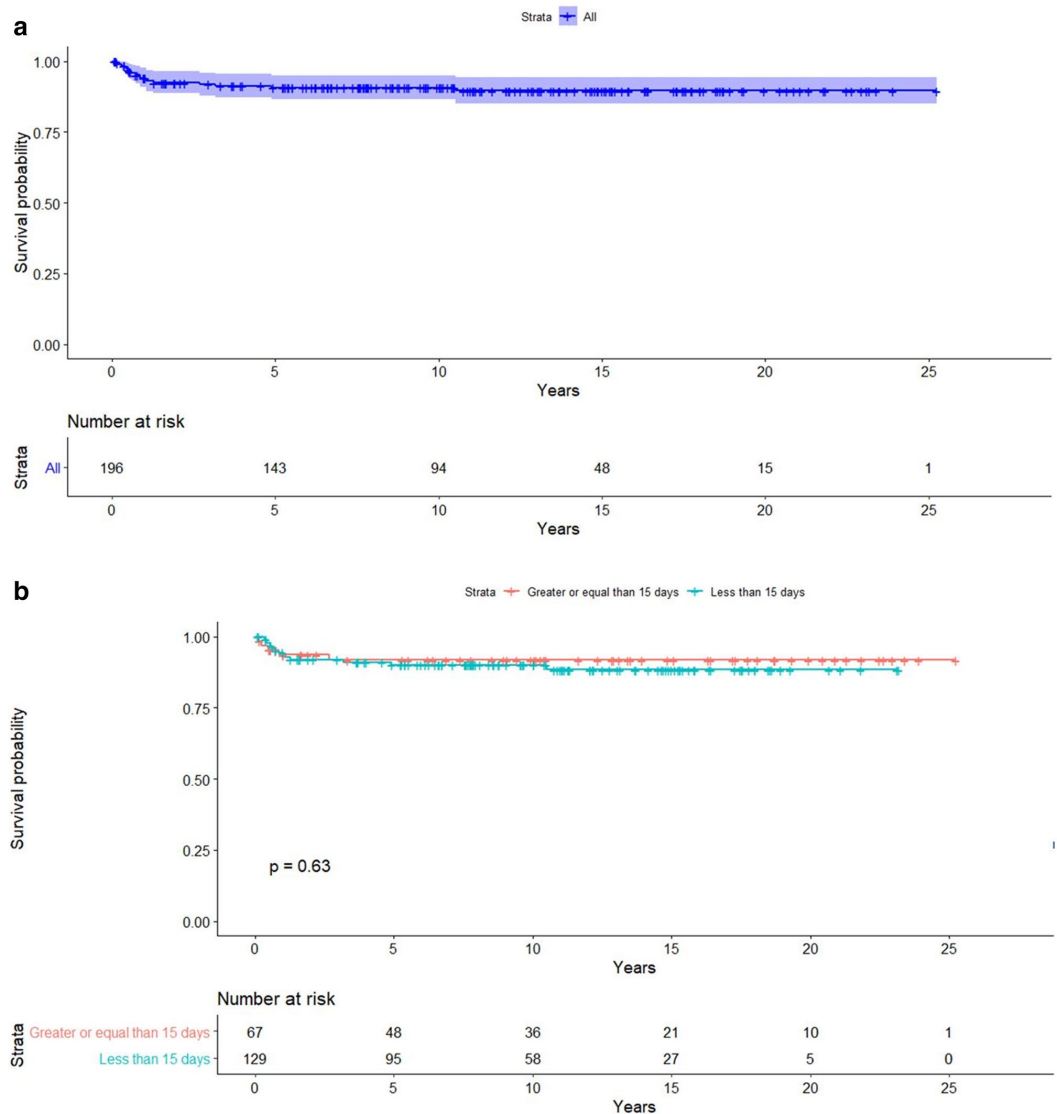
Kaplan–Meier survival plot, showing freedom from re-intervention, overall (**a**) and according to age subgroup (**b**)

Last, freedom from mortality (Fig. 1) and re-intervention on aorta (Fig. 2) were 94.3% and 96.7%, respectively, at a median FU of 10.4 years.

### Comment

In this retrospective study, repair of neonatal CoAo was performed mostly by EEEA through thoracotomy, with a very low postoperative morbidity and mortality. Also, freedom from re-intervention for late recoarctation was 96.7% at a median FU of 10.4 years, which, to the best of our knowledge, is the longest ever reported [15–17]. Our experience shows that nowadays an effective neonatal repair of CoAo has excellent long-term outcomes.

#### Surgical Technique

Currently, EEEA is considered to be the most effective and safest approach even in patients with HAA [17]. More than 80% of our patients underwent surgical repair through EEEA (Table 2). In our hands, it had a good safety profile, with an overall operative mortality as low as 2.3%, as confirmed also by other experiences [17], and with excellent late outcomes (RR 0.385, *p* = 0.048), as demonstrated by the meager re-intervention rate on the aortic arch at long-term follow-up. In our series, EEEA showed a tendency to protect from late recoarctation and late re-intervention on aortic arch (*p* = 0.09, Table 6), even if it was not confirmed after IPTW. Undoubtedly, EEEA has many advantages, such as complete resection of CoAo and ductal tissue, immediate access for hypoplasia of transverse arch, and preservation of left subclavian artery. Moreover, it does not require use of prosthetic tissue and can be performed either through left thoracotomy or sternotomy. As noted elsewhere [18], extensive proximal and distal tissue dissection are of paramount importance to mobilize the elastic neonatal aorta and brachiocephalic vessels, and to allow adequate exposure of PAA to the ascending aorta, and an effective aortic arch reconstruction without the drawbacks of CPB or DHCA. Moreover, an extensive dissection may prevent from late scar tissue retraction and can reduce recoarctation rate, and it was found to be safe, with no related complications (such as chylothorax) in our experience [8].

#### Hypoplastic Aortic Arch

Several studies reinforced the importance of identifying cut points that could help the surgeon to decide the most effective approach to achieve optimal repair with a low risk of late re-intervention for residual aortic arch hypoplasia [19, 20]. Kotany [15] reported that despite severe PAA stenosis (*z*-value < − 6), EEEA still had a 90% freedom from reoperation at 3 years. Tulzer [19] identified a PAA cut-off *z*-value of − 4.50 for a safe EEEA and a freedom from re-intervention of 90.12% at 10 years. According to Gropler [17], a *z*-value lower than − 4.1 for PAA and lower than − 2.8 for DAA may accurately predict the candidate selection for median sternotomy with a good sensitivity profile. Similarly, we collected echocardiographic measurements of PAA and DAA. However, due to insufficient data, we could not recognize useful cut-off values, since none of the 40 patients with available measurements had late recoarctation. Even if we cannot demonstrate whether hypoplastic PAA rather than DAA can cause recoarctation, in our neonatal series we had a very low re-intervention rate (9.2%) at a very long median follow-up of 10.4 years, which is longer than in other recent reports [17].

However, although minimized, the problem of recoarctation still exists. Transverse aortic arch hypoplasia and tubular hypoplasia are typically associated with intracardiac defects, such as large VSD or hypoplastic left heart syndrome. Currently, transverse aortic arch is considered of an acceptable size if its *z*-value diameter is > − 3, or if it is equal (in mm) to patient’s body weight + 1 [7]. As stated elsewhere [11], a hypoplastic PAA with *z*-score ≥ − 6 involving ascending aorta may be corrected with good results by EEEA through thoracotomy. Conversely, severe hypoplastic PAA (< − 6) with or without hypoplastic ascending aorta should be corrected with better outcomes by midline sternotomy with CPB [7], to reduce the risk of leaving a residual gradient. On this basis, we currently choose sternotomy with CPB in case of associated complex CHD, extremely hypoplastic ascending aorta and PAA (*Z*-score < − 6), or in candidates to simultaneous VSD repair.

#### Late Arterial Hypertension

Gropler et al. reported a prevalence of late hypertension of 18% [17]. Recently, Lee et al. [21] remarked a high prevalence (59%) of late-onset hypertension after CoAo repair, with 37% of patients presenting recoarctation and late hypertension (OR 2.28). In that series, most patients had repair with a left subclavian flap (41%), a prosthetic patch (11%), or simple resection (2%). Our IPTW analysis suggests that the repair technique (when well performed) may not affect significantly the onset of arterial hypertension. Age at repair is probably what makes the difference. In our series, arterial hypertension was significantly lower than other reports (9.2%). Only 18 patients had medically treated hypertension and all had a normal abdominal aorta pulsed wave Doppler ultrasound at long-term FU. Our median age at repair was 11 days, which might contribute to minimize the known risk factor for hypertension development that is older age [22].

#### Lessons from our Experience

In our experience, surgical repair of CoAo was mostly performed by the lateral approach through left thoracotomy. This approach has been favored since it avoids CPB and its related risks. This approach has been used also in cases of complex CoAo (with associated major intracardiac lesions), in the setting of a planned staged complete repair. Median sternotomy with CPB was generally preferred in case of complex CHD, with the need of multiple concomitant surgical procedures, or with severe PAA hypoplasia. This surgical strategy in our hands has been showing excellent early and late outcomes, and interestingly, we observed a low incidence of late re-interventions even when distal HAA was associated.

On this basis, and since balloon dilation was highly effective for late recoarctation treatment, we strongly advise for early CoAo repair by no-CPB approach through a left thoracotomy. Also, few patients are on anti-hypertensive therapy at follow-up, which demonstrates the effectiveness of the neonatal repair on reversing the hypertension physiopathological mechanism.

## Limitations

Although our series presents one of the longest follow-up times in patients undergoing neonatal CoAo repair, an intrinsic limitation is the retrospective nature of the investigation, which covered more than two decades of surgical experience. This allowed the collection of precise echocardiographic measures of PAA and DAA in only 40 patients. Therefore we do not have enough data to discriminate between PAA and DAA impact on late recoarctation. Last, we could not collect enough data on late term stress test to evaluate hypertensive response on effort.

## Conclusion

Surgical repair of CoAo in neonates by lateral approach without CPB is a safe and low-risk procedure, with excellent early and late outcomes. The incidence of late re-interventions is low. As the need for re-intervention on aortic arch has been rare in our series and balloon dilation was highly effective for late recoarctation treatment, neonatal repair of CoAo without CPB through a left thoracotomy is an optimal approach for a long-term effective treatment of CoAo, even when distal HAA is associated.

## Data Availability

Upon request.
